# The role of emotion in learning trustworthiness from eye-gaze: Evidence from facial electromyography

**DOI:** 10.1080/17588928.2015.1085374

**Published:** 2015-05-15

**Authors:** Luis R. Manssuer, Ralph Pawling, Amy E. Hayes, Steven P. Tipper

**Affiliations:** ^a^ School of Psychology, Bangor University, Gwynedd, UK; ^b^ School of Sport, Health and Exercise Sciences, Bangor University, Gwynedd, UK; ^c^ Department of Psychology, University of York, York, UK

**Keywords:** Gaze-cueing, Emotion, Facial EMG, Trustworthiness, Face evaluation

## Abstract

Gaze direction can be used to rapidly and reflexively lead or mislead others’ attention as to the location of important stimuli. When perception of gaze direction is congruent with the location of a target, responses are faster compared to when incongruent. Faces that consistently gaze congruently are also judged more trustworthy than faces that consistently gaze incongruently. However, it’s unclear how gaze-cues elicit changes in trust. We measured facial electromyography (EMG) during an identity-contingent gaze-cueing task to examine whether embodied emotional reactions to gaze-cues mediate trust learning. Gaze-cueing effects were found to be equivalent regardless of whether participants showed learning of trust in the expected direction or did not. In contrast, we found distinctly different patterns of EMG activity in these two populations. In a further experiment we showed the learning effects were specific to viewing faces, as no changes in liking were detected when viewing arrows that evoked similar attentional orienting responses. These findings implicate embodied emotion in learning trust from identity-contingent gaze-cueing, possibly due to the social value of shared attention or deception rather than domain-general attentional orienting.

Eye-gaze direction is a powerful cue to the object of another individual’s attention and triggers rapid and automatic attention shifts to the same location in observers. This is exemplified in studies of gaze-cueing where responses to targets presented laterally to a face are quicker when the face gazes at the target, congruently, compared to when gazing away, incongruently (Driver et al., 1999; Friesen & Kingstone, 1998; Frischen, Bayliss, & Tipper, 2007). Gaze-cues can also trigger emotional reactions. The shared attention induced by congruent gaze-cues has been shown with functional magnetic resonance imaging (fMRI) to elicit larger activity in reward-related subcortical brain regions compared to the non-shared attention induced by incongruent cues (Gordon, Eilbott, Feldman, Pelphrey, & Vander Wyk, 2013; Schilbach et al., 2010). Emotional responses may be important for learning the trustworthiness of faces when gaze-cues are contingent upon identity. Faces that consistently gaze congruently become judged more trustworthy than faces that consistently gaze incongruently (Bayliss, Griffiths, & Tipper, 2009; Bayliss & Tipper, 2006). Recently, it has been shown with EEG that the emotion-related late positive potential (LPP) component tends to increase to incongruent faces throughout the identity-contingent gaze-cueing task as trust is learned, suggesting particular sensitivity to deception (Manssuer, Roberts, & Tipper, 2015). However, while the LPP is a sensitive index of emotion processing, it cannot provide information about the role embodied emotional states play in the learning of trust. One may predict, based on embodied cognition theories and the somatic marker hypothesis, that it is not only changes in brain activity that are required but also feedback from bodily states that are part of the emotional response (Damasio, 1996; Niedenthal, 2007).

The aims of the current study were to investigate implicit positive and negative embodied emotional reactions to identity-contingent gaze-cues and their relation to trust learning using facial electromyography (EMG) recorded from the zygomaticus cheek muscle and corrugator brow muscle. Facial EMG is useful because it can be recorded continuously without the participant’s awareness that their emotional responses are being gauged, making it a valuable measure of implicit emotional responses. Our hypothesis is that for the emergence of changes in trust ratings, standard gaze-cueing effects are not sufficient. Simply being faster to respond to a target gazed at by another person does not in itself produce a change in the degree to which we trust that person. What is critical is an embodied emotional reaction to the gaze-cues elicited when default expectations that other people are helpful are violated. Hence, during incongruent trials when another person gazes away from a target, negative emotional reactions can be activated, and it is this negative emotion that is associated with the viewed face, resulting in a decrease in feelings of trust toward that face.

Different patterns of EMG activity correlate with various indexes of emotion. The contraction of the corrugator is a crucial action unit for the expression of anger and distress (Ekman & Friesen, 1978). Strong evidence for the relation of corrugator EMG activity to negative emotion is demonstrated by the reliable correlations with ratings of affect in response to affective pictures. Corrugator activity increases linearly as ratings become more negative and decreases as ratings become more positive (Lang, Greenwald, Bradley, & Hamm, 1993; Larsen, Norris, & Cacioppo, 2003), making it a useful measure of the valence of emotional reactions. It is also correlated with amygdala responses to affective pictures (Heller, Greischar, Honor, Anderle, & Davidson, 2011; Heller, Lapate, Mayer, & Davidson, 2014), a brain region that is associated with emotion, facial expression, and the processing of social-affective stimuli (Gothard, 2014; Mattavelli et al., 2014). Therefore, we predict that corrugator activity will be larger on incongruent trials, reflecting the negative emotion evoked when another person directs attention away from a relevant target object. Predictions for the zygomaticus are less clear because this muscle shows a bivalent response profile, correlating with affective ratings of both positive and negative stimuli (Lang et al., 1993; Larsen et al., 2003), and is involved in both smiling and grimacing (Bradley & Lang, 2000; Ekman & Friesen, 1978).

However, the key hypothesis is that if embodied emotional reactions mediate the learning of trust from gaze-cues, then patterns of EMG activity will differ in those people who do, and do not, show specific patterns of trust ratings at a later time. In contrast, standard gaze-cueing effects reflected in target processing reaction times will not differ in these two trust populations. These predictions are derived from the somatic marker hypothesis (Damasio, 1996), according to which decisions and choices are based on feedback from embodied emotion states. For example, in the Iowa gambling task, anticipatory skin conductance responses are enhanced when choosing from a card deck associated with disadvantageous outcomes, but only in those participants who learn a behavioral aversion to these decks (Bechara, Damasio, Tranel, & Damasio, 1997). More pertinently, Dunn and Schweitzer (2005) have shown that when participants were induced to feel angry, they trusted others less than when they were induced to feel happy, suggesting that they relied on their emotion states to inform decisions about trustworthiness.

Therefore, in order to examine if individual differences in the learning of trust relate to differences in emotional reactions measured via EMG, effects of gaze-cueing on EMG activity were compared between participants who exhibited trust effects and those who did not. At the moment of cueing, when the eyes are gazing toward or away from targets, the trust effect participants will produce increased EMG responses to incongruent cueing trials whereas the no-trust effect participants will not (see Neta, Norris, & Whalen, 2009, for similar analyses). The trial procedure also allowed us to examine whether EMG activity could index the retrieval of learned emotion states during exposure to the face before the gaze-cue. Finally, in order to determine whether the learning of evaluations is specific to the social-affective qualities of faces and gaze, in a second experiment, we examined whether liking of distinctive arrow-cues could be changed by consistently cueing congruently or incongruently. Arrows are clearly non-social but cue attention in a similar manner to gaze (Bayliss, Di Pellegrino, & Tipper, 2005; Bayliss & Tipper, 2005; Tipples, 2002, 2008).

## EXPERIMENT 1: GAZE-CUEING AND TRUSTWORTHINESS

### METHOD

#### Participants

In the gaze-cueing experiment, participants were comprised of 50 volunteers from Bangor University with a mean age of 22 years (*SD = *4) and a gender split of 25 males and 25 females. Participants were mostly right-handed (*N *= 43), neurologically normal, with normal or corrected-to-normal vision and received course credit for taking part. All procedures were approved by the Bangor University ethics committee.

#### Stimuli and apparatus

The face stimuli consisted of 16 full-color images of eight male and eight female faces with facial expressions that were mildly happy. Past research has found largest changes in trust judgments when the gaze-cueing faces express happiness (Bayliss et al., 2009), but EMG research has shown zygomaticus activity in response to happy faces (Cannon, Hayes, & Tipper, 2009; Dimberg, Thunberg, & Elmehed, 2000). Thus, a high-intensity expression would confound the measurement of responses to gaze. In addition, mildly happy faces have typically been preferred over purely neutral faces as comparison stimuli in experiments of facial expression perception due to the tendency for people to smile slightly in normal social interactions, where neutral faces would be perceived as hostile (Mattavelli et al., 2014; Phillips et al., 1998, 1999). Therefore, mildly happy faces were created by morphing a neutral version of each face with a happy version (from the NimStim face database; Tottenham et al., 2009) to create 20 frames varying from neutral to happy. A set of 10 observers were then asked to adjust each face to the point at which it could just be detected as happy. The average frame chosen was used in the experiment. The faces were matched in pairs based on gender, race, and age, and have been verified for equality in attractiveness and trustworthiness judgments in their neutral state by a set of 12 independent raters in Bayliss et al. (2009).

For each participant one of the faces in each pair was designated congruent and the other was designated incongruent. The identity of the face in each pair designated congruent or incongruent was counterbalanced across participants. There was no significant difference between counterbalancing groups in the initial difference in ratings between pairs of faces, *t*(48) = 0.063, *p *= .950, [−12.01 12.81]. Thus, at the initial ratings, incongruent and congruent faces were equal in terms of trustworthiness judgments (see Figure 2). For each face, leftward and rightward gaze-cues were created by moving the irises into the left/right-hand corners of the eyes with image editing software. Faces were presented centrally at a pixel resolution of 300 × 385. The target stimuli were a set of 32 garage and 32 kitchen objects used in past studies (Bayliss et al., 2009; Bayliss & Tipper, 2006; Manssuer et al., 2015). There were 16 unique objects in each category, which were in two different orientations (left or right mirror reversed). All were blue in color and presented at a resolution of 175 × 175 pixels centrally to the left- or right-hand side of the face in line with the eyes. The experiment was conducted using a screen resolution of 800 × 600 pixels and was run on E-Prime version 1.0.

**Figure 1.**
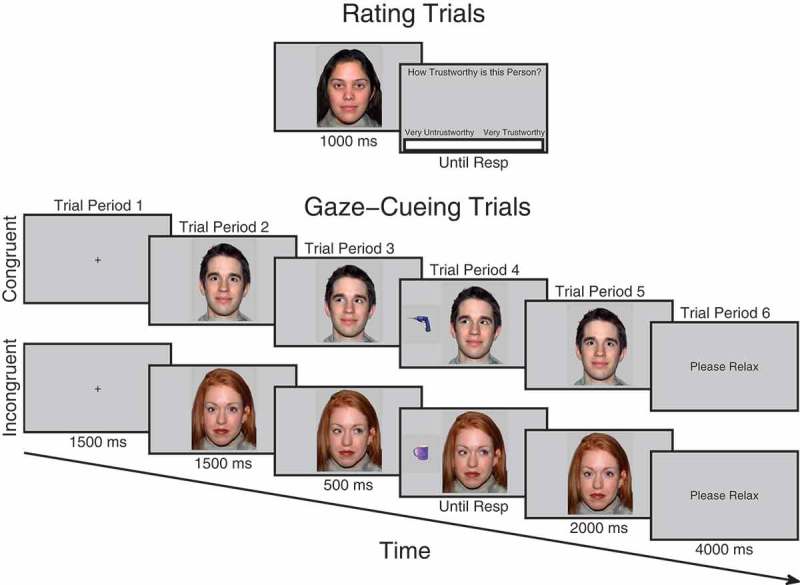
Schematic illustration of trial procedure for rating and cueing trials. On rating trials before and after cueing, participants observed each face for 1000 ms after which a visual analog rating scale appeared requiring participants to click the point on the scale that represented how trustworthy they judged the face to be. During cueing trials, participants saw a fixation cross for 1500 ms, followed by a face looking directly for 1500 ms after which it changed its gaze direction and remained for 500 ms when an object appeared to the left- or right-hand side of the face and disappeared when the participant responded which also triggered the face to look back directly at the participant for another 2000 ms. Not drawn to scale. Faces reprinted with permission from the MacArthur Network.

**Figure 2.**
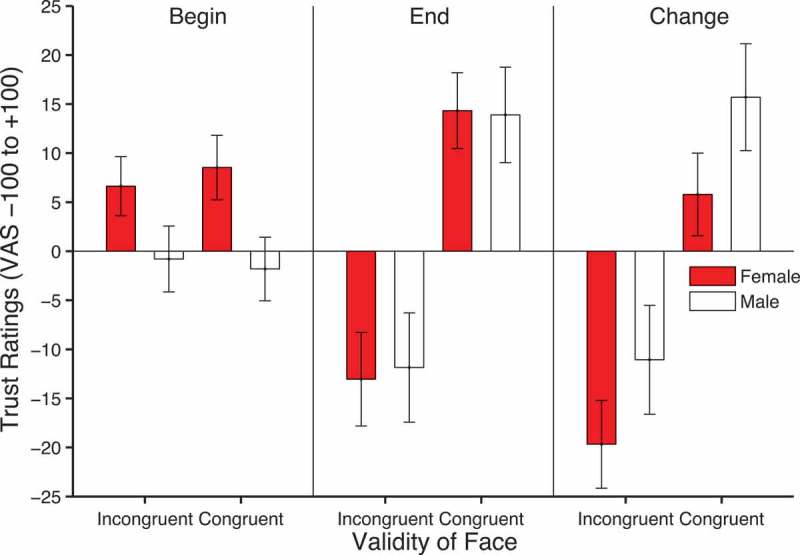
Mean trustworthiness ratings by validity and face gender, given before (left) and after (middle) cueing and the change in ratings (right), computed by subtracting beginning from end ratings. Error bars show ±1 standard error of the mean.

#### Design and procedure

Before the experiment began, participants were briefed and then presented with a slideshow of all the kitchen and garage target objects that would be presented in the experiment. They were asked to say out loud to the experimenter whether the object belonged in a kitchen or a garage, in order to check that they could perform the task correctly. The experimenter gave feedback for incorrect responses. The electrodes were then mounted on the face. Areas of the skin on the forehead, left brow, and cheek regions were cleaned using facial cleanser, alcohol swabs, and gently exfoliated with an abrasive pad before electrode gel was applied to the electrode sites. Two 4 mm silver-chloride electrode pairs were filled with conductive gel and attached to the approximate locations of the corrugator supercilii and zygomaticus major muscles using double-sided adhesive electrode cuffs (in accordance with Fridlund & Cacioppo, 1986). A ground electrode was attached to the forehead. In order to avoid demand characteristics associated with awareness that the EMG is recording emotional facial expressions, participants were told the cover story that the electrodes on the forehead were measuring frontal lobe EEG activity and that the electrodes on the cheek were reference electrodes in a face and object recognition ERP experiment (Fridlund & Cacioppo, 1986; Tassinary, Cacioppo, & Vanman, 2007). Whilst the electrodes settled onto the skin, participants completed the State-Trait Anxiety Inventory (Spielberger, 1983). Participants then began the three phases of the experiment: (1) initial trust rating phase, (2) gaze-cueing task phase, (3) final trust rating phase (see Figure 1).

#### Trustworthiness rating phases

Both beginning and end rating phases were the same (see Figure 1). The purpose of the beginning rating was to provide an initial baseline measure of trust before gaze-cueing. This helps control for any initial variability in trust that is not due to gaze-cueing and which could potentially bias a particular condition. Each trial began when participants pressed the spacebar, at which point a fixation cross appeared for 1000 ms, followed by a directly gazing face for 1000 ms, and then a screen containing a visual analog rating scale (VAS) asking “*How trustworthy is this person?*” At this point, a cursor was visible on the screen and participants used the mouse to click along the scale at the point that represented how trustworthy they judged that person to be. The extreme left of the scale was labeled “*Very* *Untrustworthy*” and the extreme right of the scale was labeled “*Very Trustworthy*.” The center of the screen therefore represented neutral. When participants clicked on the scale, the computer recorded a trust rating between −100 and +100. The order of face identity on each trial was randomized.

#### Gaze-cueing phase

In the gaze-cueing phase (see Figure 1), participants initiated each trial with the spacebar. A fixation cross appeared for 1500 ms followed by a directly gazing face for 1500 ms. The face then changed gaze direction and remained gazing in that direction for 500 ms after which an object appeared to the left- or right-hand side of the screen next to the face. The face stayed presented on the screen during object presentation. The object disappeared as soon as the participant made a response, or after five seconds had elapsed. At the same time as the object disappeared, the face gazed directly again for 2000 ms. The participant was then presented with a text screen saying “*Please Relax*” for 4000 ms. Participants were told that their task was to classify the object as to whether it belonged in a kitchen or a garage as quickly but as accurately as possible and that the face was irrelevant to their task. One of each matched face pair consistently gazed toward the target object, congruently, whereas the other always gazed away, incongruently. There were five blocks in total, each comprising 32 trials. In each block, each of the 16 faces was presented on two trials, once gazing leftward and once gazing rightward. Trial orders were randomized within each block. Objects within each category were randomly sampled without repetition except when in a different orientation. Responses were counterbalanced, as half of the participants were instructed to press the *H* button if the object belonged to a garage and the spacebar if it belonged to a kitchen, whereas the other half were instructed to do vice versa. Participants responded with their index finger on the *H* button and thumb on the spacebar. If no response was made or if the response was incorrect, an error tone was sounded lasting 1000 ms. Participants completed eight practice trials beforehand with faces that were not used in the main experiment. After the gaze-cueing phase was complete, the session then finished with the participants completing the trust rating phase again after which the electrodes were removed and the participants were debriefed.

#### Electromyographic recording

Electromyography was recorded using a Biopac MP100 system with two EMG100C amplifiers. All data were sampled at 2 kHz, amplified by a factor of 5000 and filtered online with a high pass filter of 10 Hz, a low pass filter of 500 Hz and a notch filter at 50 Hz. Subsequently, the data were band pass filtered between 20–400 Hz which has been shown to be the optimal bandwidth for the removal of artifacts such as eye-movements, eye-blinks, and brain activity from facial EMG (Van Boxtel, 2001). As EMG data are bipolar voltages around zero, the data were full-wave rectified to measure amplitude. The time courses of each individual’s EMG activity across all trials were also inspected visually whilst blind to conditions. Separate sets of artifact trials were removed for the corrugator and zygomaticus muscles. Trials containing large inflections caused by non-expressive movements, such as yawns or coughs, were removed. Activity in all trial periods was divided by the mean activity between 900–1400 ms of the fixation period of each trial. This baseline correction controls for the fluctuations and noise in the EMG signal that varies over the course of the experiment. EMG activity is therefore expressed as a ratio of muscle activity with respect to baseline. The data were pre-processed in Matlab 2014a, averaged into 100-ms time bins and statistically analyzed in SPSS version 20. These are all common practices in EMG data processing (Cannon et al., 2009; Fridlund & Cacioppo, 1986; Larsen et al., 2003; Moody, McIntosh, Mann, & Weisser, 2007; Winkielman & Cacioppo, 2001).

#### Data screening protocol and analysis

Table 1 shows the mean percentages of trials removed from analyses. All trials on which participants made an error or did not respond (*M *= 2.51%, *SD* = 1.95% of trials) were removed from reaction time and EMG analyses along with trials with reaction times above or below two standard deviations from each participant’s mean (*M *= 4.31%, *SD *= 1.34% of trials). In addition, any remaining reaction times larger than 1500 ms were removed (*M *= 1.95%, *SD *= 3.33% of trials), a criterion typically employed in gaze-cueing studies (Langton & Bruce, 1999; Teufel, Alexis, Clayton, & Davis, 2010). The first trial of the experiment and trials immediately succeeding error trials were removed from the EMG data to eliminate facial reactions to novelty and errors (*M *= 2.93%, *SD *= 1.65% of trials). Trials with artifacts observed were removed from the corrugator (*M *= 5.28% of trials, *SD *= 2.98%) and zygomaticus (*M *= 8.6% of trials, *SD *= 3.7%) analyses separately. Paired samples *t*-tests were used to test for differences in the number of errors, outliers and artifacts between incongruent and congruent conditions. There were no significant differences in outliers, *t*(49) = −1.02, *p* = .313, 95% CIs [-.71, .23], corrugator artifacts, *t*(49) = -0.594, *p* = .555, 95% CIs [-.55, .37], zygomaticus artifacts, *t*(49) = -0.375, *p* = .709, 95% CIs [-.79, .54], and errors, outliers, and corrugator artifacts combined, *t*(49) = −1.85, *p* = .071, 95% CIs [−1.46, .063] or errors, outliers, and zygomaticus artifacts combined, *t*(49) = −1.64, *p* = .108, 95% CIs [−1.56, .16]. Errors were significantly more frequent on incongruent trials, *t*(49) = −2.04, *p* = .047, 95% CIs [-.671, -.004] (see Table 1), supporting the longer reaction times on such trials.

**TABLE 1:** Mean percentage of trials containing errors, outliers, and artifacts across conditions in experiment 1

	Errors	Outliers	Corrugator artifacts	Zygomaticus artifacts
Congruent	1.09%	3.01%	2.58%	4.24%
Incongruent	1.43%	3.25%	2.70%	4.36%

In order to divide participants into those showing trust effects and those who did not, each participant’s mean rating change score (end—beginning) for congruent and incongruent faces were calculated and graphed. Those who showed a larger negative change to incongruent compared to congruent, without overlap in the standard error bars, were classified as showing a trust effect (referred to in between-subjects analyses as the trust effect group), whereas those who showed no difference or changes in the opposite direction were classified as not showing a trust effect (referred to as the no-trust effect group). This was then entered into the reaction time and EMG analyses of variance (ANOVAs) as a between-subjects factor. Overall, 27 participants showed trust effects whereas 23 participants did not. All analyses of between-subjects effects were reproduced with participant gender as the between-subjects factor. As no effects of participant gender were significant we refrain from reporting them here. ^1^ ^1^It is noteworthy that there were no participant sex differences in gaze-cueing. This contrasts with Bayliss et al. (2005), where female participants produced larger gaze-cueing effects than males at longer stimulus onset asynchronies. However, in Bayliss et al. (2005) participants viewed two cartoon images, one of a male and one of a female face. These faces were not lifelike, and not relevant to the target detection task. Hence, the irrelevant nature of the stimulus facilitated the detection of the subtle differences between men and women’s response to gaze-cues. In contrast, in the current study a range of real male and female face images were presented. Furthermore, at the start of the study these faces were rated for trustworthiness. This increased the salience of the faces for all participants, diminishing the contrast between male and female participants and increasing the salience of male and female images in trust ratings.


For analysis of the EMG data in trial period 4, where the duration depends upon the reaction time, the trial period was cut down to a maximum duration of 1000 ms as this is roughly equal to the mean reaction time +1 standard deviation.

### RESULTS AND DISCUSSION

#### Evaluations of trustworthiness

Trust ratings were analyzed using a 2 × 2 × 2 within-subjects ANOVA with factors of validity, face gender, and time of rating. There was a significant effect of validity, *F*(1, 49) = 11.12, *p *= .002, *η* *_p_* ^2^ ^2^Anxiety data were collected to rule out the possibility that the effects could also be explained by individual differences in this variable. = .19, but more importantly, a significant validity × time of rating interaction, *F*(1, 49) = 12.895, *p *= .001, *η_p_* ^2^= .208. As shown in Figure 2, these effects are clearly driven by the higher trust ratings for congruent (*M = *14.108, *SEM = *3.74) compared to incongruent faces (*M = −1*2.45, *SEM =* 4.65) in the final rating phase. There was also a significant interaction between time and face gender owing to higher trust ratings for females (*M* = 7.585, *SEM* = 2.731) compared to males (*M* = −1.297, *SEM* = 2.813) in the initial rating phase compared to the final rating phase, *F*(1, 49) = 7.352, *p* = .009, *η_p_* ^2^ = .130. No other effects reached significance. To formally identify whether the sources of the main effects and interactions described above occurred before or after cueing, 2 × 2 within-subjects ANOVAs with factors of validity and face gender were run on the beginning and end ratings separately. These analyses showed that, in the initial ratings, there was a significant effect of face gender, *F*(1, 49) = 8.46, *p* = .005, *η_p_* ^2^ = .147, but no significant effect of validity, *F*(1, 49) = 0.029, *p* = .865, *η_p_* ^2^ = .001, and no validity × face gender interaction, *F*(1, 49) = 0.493, *p* = .486, *η_p_* ^2^ = .01. In contrast, after cueing, there was a significant effect of validity, *F*(1, 49) = 13.397, *p* = .001, *η_p_* ^2^ = .215, but no significant effect of face gender, *F*(1, 49) = 0.012, *p* = .914, *η_p_* ^2^ = .000, and no validity × face gender interaction, *F*(1, 49) = 0.071, *p* = .791, *η_p_* ^2^ = .001.

The effects of gaze-cues on changes in trust confirms previous findings (Bayliss et al., 2009; Bayliss & Tipper, 2006; Rogers et al., 2014). However, the current findings go beyond previous work in two ways. First, we have employed an initial baseline measure of trust allowing us to observe the direction of the effect. It was possible that congruent faces could increase in trustworthiness with no effect for incongruent faces, incongruent faces could decrease in trustworthiness with no effect for congruent faces, or that both congruent and incongruent faces would show changes in trustworthiness in opposite directions. The data support the latter of these alternatives. The second new finding concerns stimulus gender differences. The positive change in trust ratings in response to congruent cues were greatest for male faces whereas the negative change in response to incongruent cues were larger for female faces. This is consistent with the idea that learning is greater when the gaze-cues are mismatched with the kinds of social interaction expected from the visual appearance of the face. Indeed, Bayliss et al. (2009) only found trust effects with smiling and not frowning faces, where the former would be initially rated as more trustworthy (Oosterhof & Todorov, 2008). This type of learning is akin to the Rescorla and Wagner (1972) learning rule, where learning is greatest when there is a discrepancy between expected and actual events.

Although analysis of all participants has clearly replicated the effects of gaze-cueing contingencies on trust assessments, not all participants showed the trust effect, and the key question is what discriminates between people who do and do not show trust effects. Our main hypothesis is that it is not just the facilitation effects of gaze-cueing on target processing that is driving the trust effect. Rather, it is an emotional signal evoked during cueing that is necessary for learning, which we expect to detect via EMG, and which may vary across participants. Therefore, for all further analyses of reaction times and EMG activity, participants were divided into two groups. One group consisted of those who showed a trust effect whereas the other group did not.

#### Gaze-cueing reaction times

**Figure 3.**
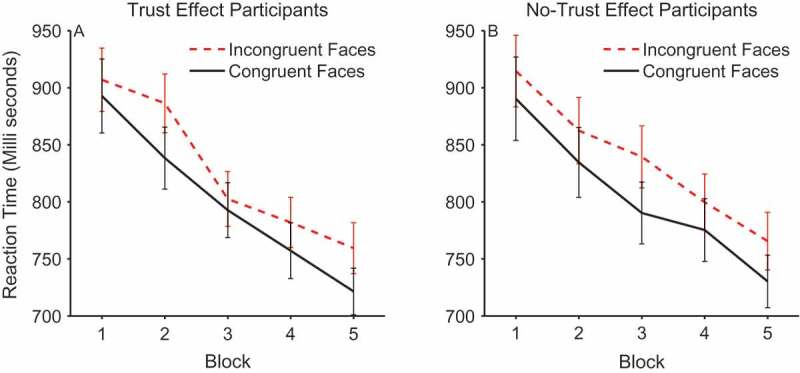
Mean reaction times on congruent and incongruent trials across blocks within participants who did (left panel) and did not show trust effects (right panel). Error bars show +/-1 standard error of the mean.

The reaction time data were analyzed using a 2 × 2 × 2 × 5 mixed design ANOVA with the between-subjects factor of trust effect and within-subjects factors of validity, face gender, and block. On average, participants’ reaction times were quicker on congruent trials (*M =* 802.28, *SEM = *17.68) compared to incongruent trials (*M = *831.85, *SEM = *16.74) and this effect of validity was significant, *F*(1, 48) = 35.038, *p *< .0001, *η_p_* ^2^ = .422 (see Figure 3). There was also a significant effect of block, which was due to a linear trend for a decrease in reaction times over the course of the experiment, *F*(1, 48) = 148.497, *p < *.0001, *η_p_* ^2^ = .756. However, there was no validity × block interaction, *F*(4, 192) = 0.840, *p *= .502, *η_p_* ^2^ = .017, suggesting that participants were not using the identity and initial gaze direction of the face to anticipate the location of the target. Importantly, there was no main effect of trust versus no-trust group, *F*(1, 48) = 2.04, *p *= .160, *η_p_* ^2^ = .041, and no interaction between trust group and validity, *F*(1, 48) = 0.009, *p *= .926, *η_p_* ^2^ = .000. Separate analysis of each group confirmed significant effects of validity within both the trust group, *F*(1, 26) = 16.23, *p* < .0001, *η_p_* ^2^ = .384, and the no-trust group, *F*(1, 22) = 20.916, *p *< .0001, *η_p_* ^2^ = .487. In order to rule out the possibility that the effects of validity described above may be due to a speed-accuracy trade-off, the same 2 × 2 × 2 × 5 ANOVA was run on the reaction times weighted by proportion of correct responses in each condition. This analysis showed that the effect of validity, *F*(1, 48) = 16.067, *p *< .0001, *η_p_* ^2^ = .251, and block, *F*(1, 48) = 95.582, *p < *.0001, *η_p_* ^2^ = .668, was still highly significant when accuracy was controlled for. No other effects reached significance.

The main finding of interest was that the cueing effects of gaze direction were the same for individuals who later showed a trust effect in the ratings and those who did not show the standard trust effect. This lack of difference in orienting of attention is in line with our hypothesis that the change in trust evoked by congruent or incongruent gaze is not simply due to attentional facilitation effects. Rather, we hypothesize that emotional reactions to the congruency of the gaze are necessary for changes in trust. That is, during interaction with the face, when the eyes look toward and facilitate target processing or look away and impair target processing, this evokes an emotional response in some participants. It is this emotional signal that is associated with the viewed face, influencing later judgments of trust and which may manifest itself in terms of a facial expression, measurable with EMG.

#### Facial electromyography results

**Figure 4.**
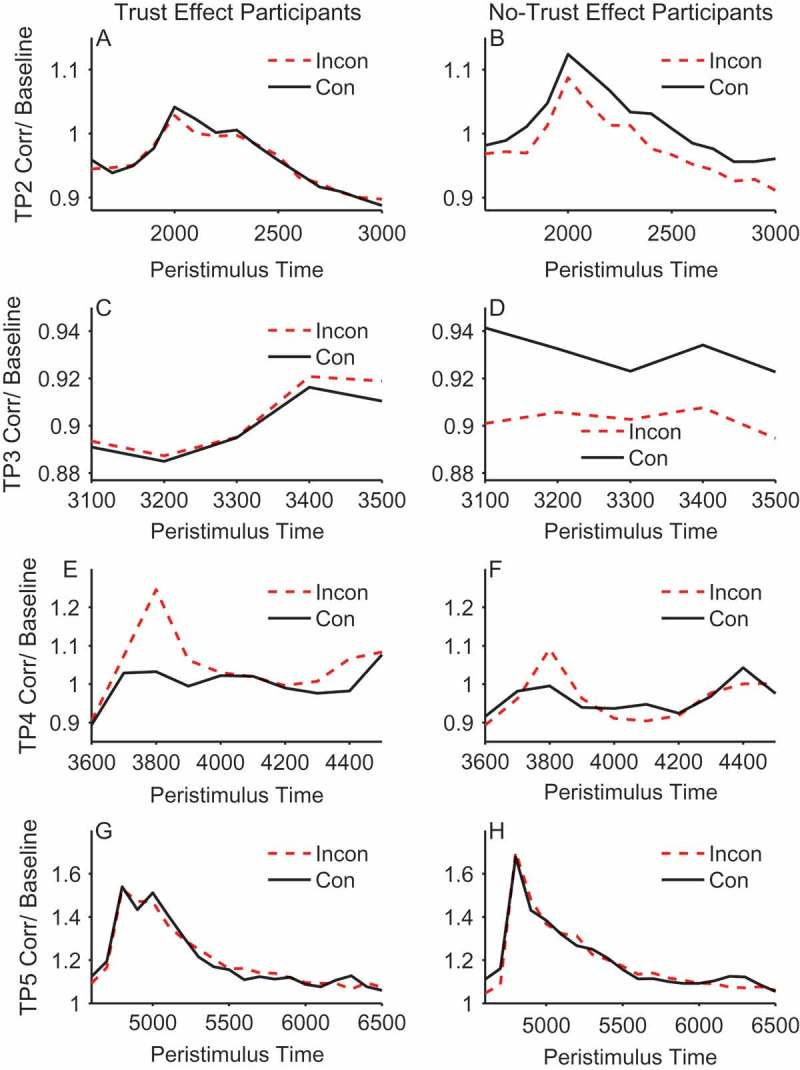
Mean stimulus-locked corrugator activity on congruent (solid line) and incongruent trials (dashed line) for trust effect (left panels) and no-trust effect (right panels) participants across trial periods 2, 3, 4, and 5 (rows). EMG units on the *y*-axis represent the ratio of activity relative to baseline (fixation). Error bars show ±1 standard error of the mean.

**Figure 5.**
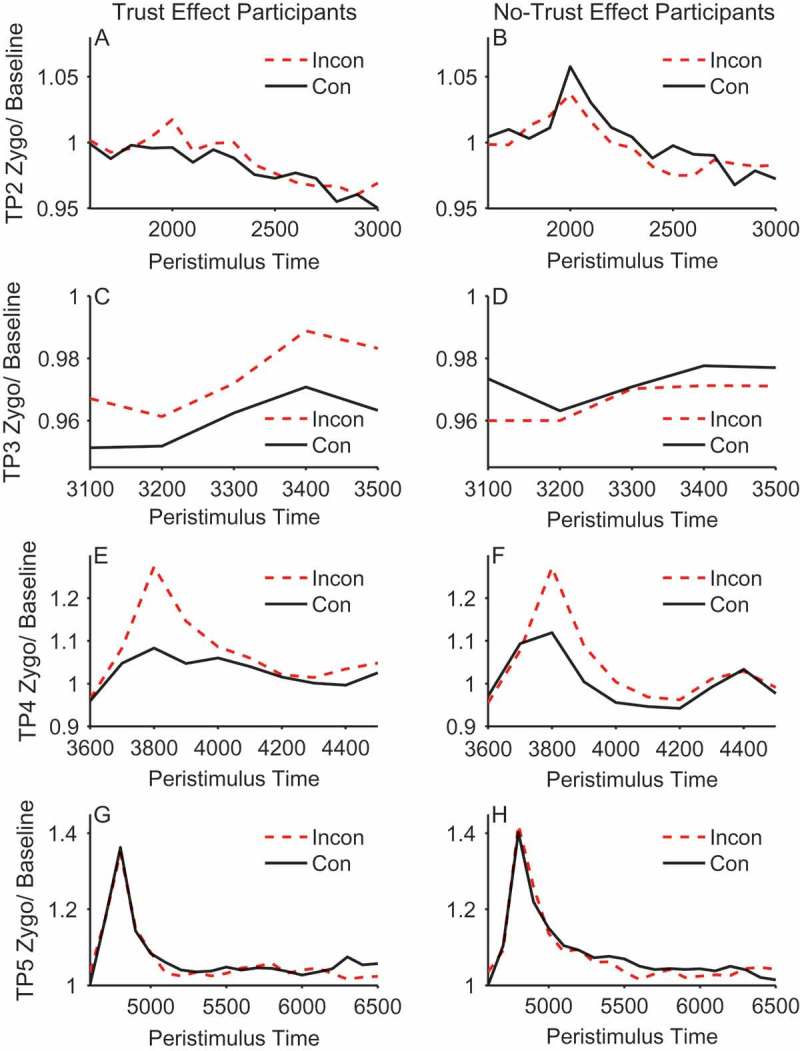
Mean stimulus-locked zygomaticus activity on congruent (solid line) and incongruent trials (dashed line) for trust effect (left panels) and no-trust effect participants (right panels) across trial periods 2, 3,4, and 5 (rows). EMG units on the *y*-axis represent the ratio of activity relative to baseline (fixation). Error bars show ±1 standard error of the mean.

**Figure 6.**
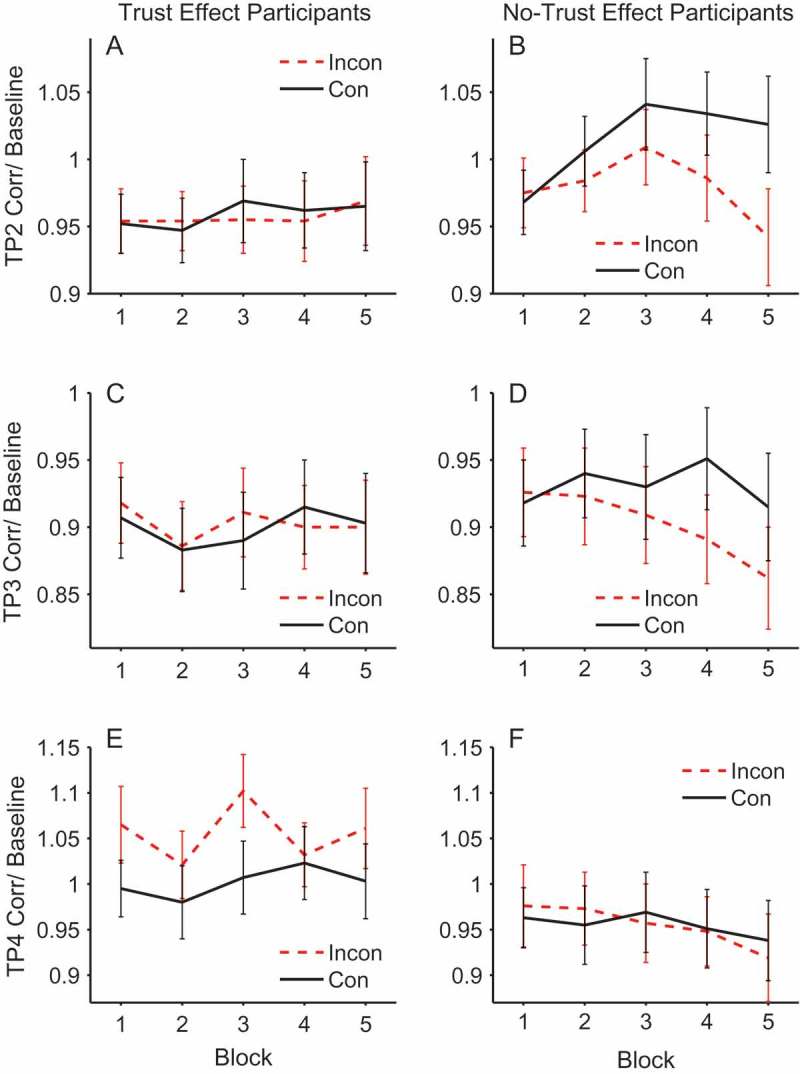
Mean corrugator activity across blocks for trust effect participants (left panels) and no-trust effect participants (right panels) in trial period 2 (top), trial period 3 (middle) and trial period 4 (bottom). Dashed lines represent incongruent and solid lines congruent. EMG units on the *y*-axis represent the ratio of activity relative to baseline (fixation). Error bars show ±1 standard error of the mean.

Figure 4 (corrugator) and Figure 5 (zygomaticus) show the EMG activity throughout the critical trial periods of 2–5 where the face is presented and gaze-cueing takes place. Hence, in the EMG analysis we focus on the individual trial periods of 2–5 when the face was visible. During these periods we examine the effects of cueing on facial muscle reactions and how this might differ in people who demonstrate our expected pattern of trust ratings (increasing trust of faces that look toward targets and decreasing trust of faces that look away from targets) as compared to those who show no differences or trends in the opposite direction. It should be noted that trial periods 2 and 3 occur before the gaze-cue and target are presented and so any contrasts between congruent and incongruent faces must be due to learning via previous exposure to the face identity-contingent gaze-cues. This means that in trial periods 2 and 3 of Block 1, there will not have been an opportunity to learn whether the face is congruent or incongruent on the first trial it is observed. Therefore, we would not expect to see a difference between validity conditions in trial periods 2 and 3 of Block 1 (see Figure 6, Panels A–D for confirmation of this). As such, Block 1 was omitted from the analysis of trial periods 2 and 3 to retain sensitivity to effects of validity. These trial periods were therefore analyzed separately within each muscle with a 2 × 2 × 2 × 4 mixed ANOVA, with the between-subjects factor of trust effect and within-subjects factors of validity, face gender, and block (2–5). In contrast, because trial periods 4 and 5 occur during/after the gaze-cue and target presentation, they were analyzed with all five blocks included in a 2 × 2 × 2 × 5 mixed ANOVA. Interactions with trust effect were followed up with separate within-subjects ANOVAs in each group.

#### Trial period 2

At this point in the trial the face is initially presented looking directly toward the participant (see Figure 1). This analysis showed that there was a significant main effect of validity in the corrugator, *F*(1, 48) = 5.478, *p *= .023, *η_p_* ^2^ = .102, but not in the zygomaticus, *F*(1, 48) = 0.146, *p *= .704, *η_p_* ^2^ = .003. The main effect in the corrugator was due to the no-trust effect participants showing a larger response to congruent compared to incongruent faces, as shown by the significant interaction between trust effect and validity, *F*(1, 48) = 4.160, *p *= .047, *η_p_* ^2^ = .080. ANOVAs conducted for each group separately showed the effect of validity was significant in the no-trust effect participants, *F*(1, 22) = 6.95, *p *= .015, *η_p_* ^2^ = .24, but was not significant in the trust effect participants, *F*(1, 26) = 0.064, *p *= .802, *η_p_* ^2^ = .002. No other effects or interactions were significant (see Figure 6, Panels A & B).

#### Trial period 3

During trial period 3 the face gazed to the left or right prior to the appearance of the target. This was a relatively brief period of 500 ms. As in trial period 2, there were no significant effects for the zygomaticus, but for the corrugator there was a significant interaction between trust effect and validity, *F*(1, 48) = 4.407, *p *= .041, *η_p_* ^2^ = .084, which was again due to the no-trust effect participants showing a larger response to congruent compared to incongruent faces, *F*(1, 22) = 5.34, *p *= .031, *η_p_* ^2^ = .195, and the absence of such an effect of validity in the trust effect participants, *F*(1, 26) = 0.031, *p *= .861, *η_p_* ^2^ = .001. No other effects or interactions were significant (see Figure 6, Panels C & D).

The results in trial periods 2 and 3 are intriguing for three reasons. First, they demonstrate retrieval of prior face congruency. That is, the face is either looking straight ahead (trial period 2) or has made a gaze shift with no target present (trial period 3). Hence, discrimination of gaze congruency must be based on previous episodes with the faces and not on the current perceptual experience. Second, the individuals who do not show the expected change in trust ratings are those who appear to be retrieving prior episodes. And third, the corrugator, which reflects a negative emotional response, is more active for congruent faces that are about to look toward the target. These somewhat unexpected observations are discussed later.

#### Trial period 4

During trial period 4 the face is gazing to the left or right and the kitchen or garage target object is presented for speeded classification. Therefore, this is the period during which the participant experiences congruent and incongruent gaze-cueing. During this gaze-cueing episode there was a marginal main effect of validity in the corrugator, *F*(1, 48) = 3.96, *p *= .052, *η_p_* ^2^ = .076, and a significant effect in the zygomaticus, *F*(1, 48) = 11.0, *p *= .002, *η_p_* ^2^ = .186, owing to a larger response to incongruent compared to congruent gaze-cues. In both cases, muscle activity peaked at 200–300 ms after target stimulus onset.

In addition, and of central importance to our hypothesis, for the corrugator within trial period 4, there was a significant interaction between trust effect and validity, *F*(1, 48) = 4.21, *p *= .046, *η_p_* ^2^ = .081. Separate ANOVAs in each group revealed a significant difference between congruent and incongruent cueing trials in those individuals who showed a trust effect, *F*(1, 26) = 6.48, *p* < .017, *η_p_* ^2^ = .200, and the absence of a significant difference in those who did not produce trust effects, *F*(1, 22) = 0.003, *p* = .955, *η_p_* ^2^ = .000. In contrast to trial periods 2 and 3, the effect was driven by a larger reaction to incongruent compared to congruent in the trust effect compared to the no-trust participants. In the zygomaticus there was no interaction between trust effect and validity, *F*(1, 48) = 0.341, *p *= .562, *η_p_* ^2^ = .186 (see Figure 6, Panels E & F).

The EMG activity in trial period 4 conforms to our predictions, at least in the corrugator. Participants who later rate faces that consistently looked toward targets as more trustworthy, and faces that looked away from targets as less trustworthy, showed greater overall activity of the corrugator to incongruent than congruent trials. Hence, this embodied negative emotional reaction when a face looks away from a target appears to be important for later trust ratings. Note, however, that this trial period 4 pattern contrasts with that of trial periods 2 and 3, where the no-trust individuals discriminated congruent from incongruent faces.

#### Trial period 5

During trial period 5 the gaze has returned to the center and participants passively view the face after response to the target. Analysis of both the corrugator and zygomaticus revealed no significant effects.

Finally, despite several novel findings, there are some potential technical issues in the current study that should be considered. The difference in the amplitude of the EMG responses to gaze-cues is unlikely to be explained as activity generated by concomitant eye-movements. Although previous studies have shown that gaze-cues can trigger micro saccades in the direction of the gaze, the changes in eye-position are small (Deaner & Platt, 2003). Larger, overt eye-movements are required to fixate the object beside the face and this is required for both congruent and incongruent cueing. Furthermore, the differential amplification of the bipolar electrode montage has the effect of canceling out common signals from distant sources and the electrodes are placed away from the optimal sites used to record lateral eye-movements. Eye blinks are unlikely to explain the effects as such artifacts occur at a frequency resolution below the threshold of the high pass filter (~20 Hz). It is also unlikely that the differences are due to larger errors for incongruent compared to congruent cues. The overall differences in the number of error trials between conditions were small (<1) and were removed from all analyses. In addition, the errors appeared to be equally distributed across all 16 identities of the face.

#### Individual differences in anxiety

Anxiety data were collected for 49 of the participants whilst the electrodes settled onto their skin. Using independent samples *t*-tests, there was no significant differences between trust effect and no-trust effect participants in terms of trait, *t*(47) = 0.783, *p *= .438, 95% CIs [−3.86, 8.87], or state anxiety, *t*(47) = 0.142, *p *= .888, 95% CIs [−4.83 5.56]. This is somewhat surprising, as it might have been predicted that individuals scoring higher on anxiety would be more sensitive to learning the contingencies between identity and gaze.^2^

## EXPERIMENT 2: ARROW-CUEING AND LIKING

Experiment 1 demonstrated that trust judgments of task-irrelevant faces can be learned from identity-contingent gaze-cues and that this appears to be mediated by individual differences in embodied emotional reactions. However, we do not know whether this effect of identity-contingent attentional-cueing on evaluations generalizes to other stimuli. Therefore, Experiment 2 attempted to replicate Experiment 1 using arrow-cues that all have individual identities, possessing complex and unique color patterns. Arrow-cues were used because there is a well-established literature demonstrating that these stimuli cue attention in the same manner to gaze (Bayliss et al., 2005; Bayliss & Tipper, 2005; Tipples, 2002, 2008). While it would be possible to use stimuli that were more similar to eyes to cue attention this could potentially risk losing cueing effects, which are key for our hypotheses. For example, Ristic and Kingstone (2005) found that circles with black dots in the center, that shifted to the left or the right, produced a cueing effect when perceived as eyes in a schematic face but not when perceived as the wheels of a car. Thus, equating the gaze- and non-gaze-cueing stimuli for perceptual properties could risk losing effects in the latter. Indeed, the power of such small deviations in gaze to cue attention may well be due to the mental state information it provides rather than just perceptual properties (Teufel et al., 2010). In Experiment 2, some arrows always pointed toward subsequent targets, facilitating responses, while other arrows always pointed away from subsequent targets, impairing responses. If the effect of gaze-cues on trust were simply due to domain-general attentional-cueing, we would expect similar effects with arrows, with incongruent arrows becoming less liked than congruent arrows.

### METHODS

#### Participants

There were 31 volunteer participants from Bangor University of which 29 were right-handed and 21 were female. Participants had a mean age of 20 (*SD *= 3.7), were neurologically normal with normal or corrected-to-normal vision, and received course credit for taking part. All procedures were approved by the Bangor University ethics committee.

#### Stimuli and apparatus

**Figure 7.**
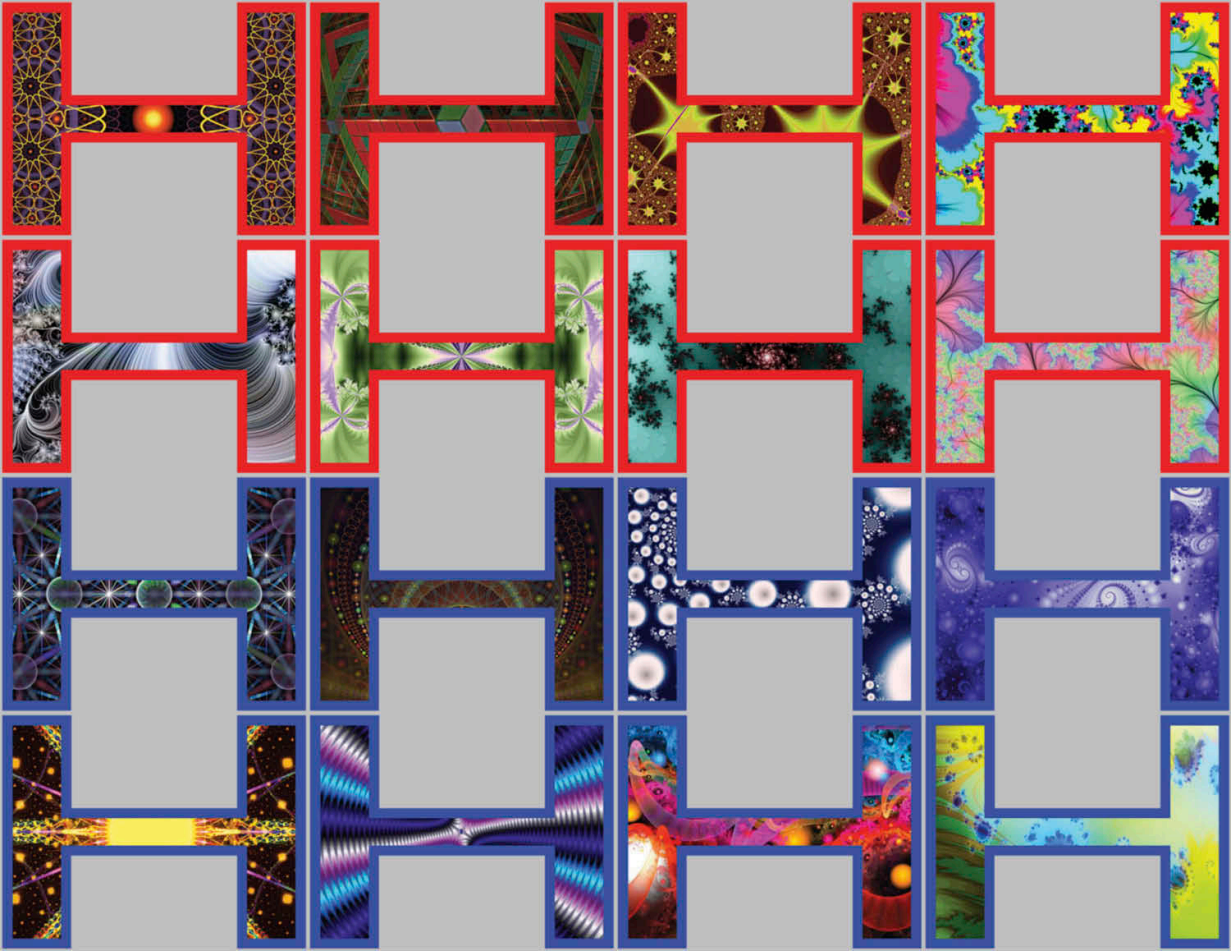
The 16 arrows in their neutral state used in experiment 2.

**Figure 8.**
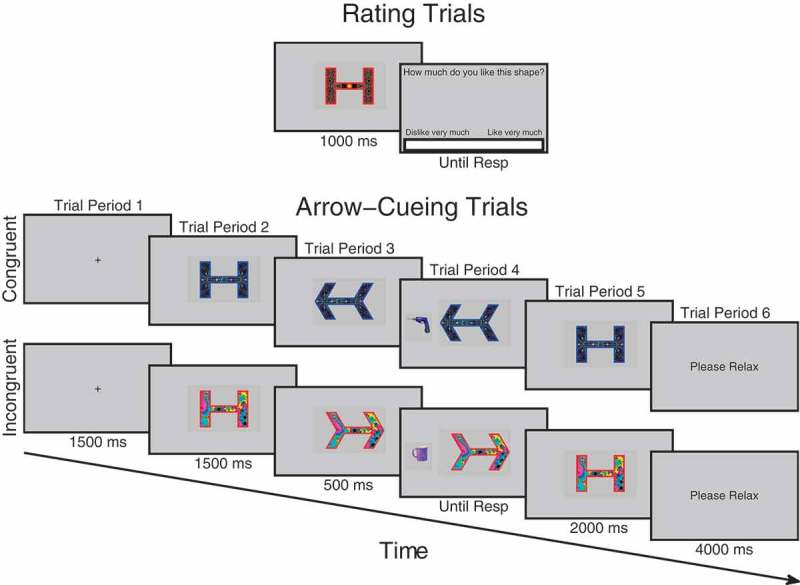
Trial procedure on rating and cueing trials for experiment 2 using arrows. On rating trials before and after cueing, participants observed each arrow/shape for 1000 ms after which a visual analog rating scale appeared requiring participants to click the point on the scale that represented how much they liked the arrow/shape. During cueing trials, participants saw a fixation cross for 1500 ms, followed by an arrow/shape in its neutral state for 1500 ms, after which it changed shape to point to the left or right for 500 ms when an object appeared to the left- or right-hand side and disappeared when the participant responded. This also triggered the arrow to go back to its neutral state for another 2000 ms.

There were 16 distinct arrows. Each arrow consisted of a head, shaft, and tail. Half of the arrows had a blue outline and the other half had a red outline. This difference in color was used to create a category that would equate face gender. Within the outline, each arrow had a distinctive fractal pattern to make them unique and discernable (see Figure 7). When in their neutral state, the head and tail of the arrow remained straight. This was to equate the directly gazing face. Directional cues were created by bending the head and tail about the shaft to the left or right by 30**°** (see Figures 7 & 8). The size of the arrows, in their neutral state, was 454 × 351 pixels. The objects were the same as in Experiment 1, except that they were enlarged to 350 × 263 pixels to be used on a 24” Samsung SyncMaster BX2431 LED display with 500 Hz refresh rate and 1920 × 1080 pixel resolution. This screen was 569 × 342 mm in dimensions. The difference between faces and arrows is that the boundary of the arrows shifts toward the objects whereas it is the eyes in the center of the face for faces. Therefore, we intentionally used a wider screen for the arrow-cueing experiment to account for the change in width of the arrows. During cueing, the point of the arrow were approximately 7.84 cm distance from the target objects whereas, in comparison, the corners of the eyes in Experiment 1 were approximately 7.38 cm distant from the target objects. In trial periods 3 and 4 when the arrow shifted at an angle of 30**°** to the left or right, the point of the arrow was 2.8 cm distance from the outer edge of the shaft when in its neutral position in trial periods 2 and 5 (see Figure 8). The arrows were not matched for visual appearance. However, counterbalancing across participants ensured that all stimuli occurred equally as frequently in the congruent and incongruent conditions. Also, taking ratings at the beginning of the experiment allowed for control of pre-existing differences between stimuli in each condition in each participant. Finally, previous data confirmed that these arrows were as discriminable as faces in a visual search task and they were equally well remembered in a subsequent recognition task.

#### Design and procedure

The design and procedure of Experiment 2 was exactly the same as Experiment 1 apart from that the cueing stimuli used were arrows instead of faces/gaze and the ratings given were on a visual analog scale of liking instead of trust (see Figure 8). Participants were asked “*How much do you like this shape?*” The rating scale ranged from “*Dislike very* *much*” to “*Like very* *much*” at the extreme left- and right-hand sides of the scale, respectivel*y*.

#### Data screening

The reaction time data were processed in the same manner as Experiment 1. Paired samples *t*-tests showed that there was no significant difference between congruent and incongruent conditions in terms of the number of errors, *t*(30) = 0.000, *p *= 1.0, 95% CIs [−1.62 1.62] and outliers, *t*(30) = 0.924, *p *= .621, 95% CIs [−2.77 1.68] (see Table 2).

**TABLE 2:** Mean percentage of trials containing errors and outliers across conditions in experiment 2

	Errors	Outliers
Congruent	3.85%	5.87%
Incongruent	3.85%	5.32%

#### Analyses

As arrows were red or blue to match the gender dimension of female and male faces, we excluded this factor from all ANOVAs as it was not of primary interest. Due to the low numbers of male participants, the participant gender factor was also not analyzed. Analogous to Experiment 1, participants were divided into those showing a liking effect related to arrow validity and those who did not. However, only five participants showed a liking effect (i.e., a larger negative change in liking for incongruent compared to congruent without overlap in the error bars), with the remaining 26 participants not showing a liking effect. Due to the small numbers of participants showing a liking effect, this factor was excluded from all ANOVAs. Moreover, the EMG data were not analyzed, as there were too few participants showing a liking effect to produce meaningful comparisons with those who did not.

### RESULTS

#### Evaluations of liking

**Figure 9.**
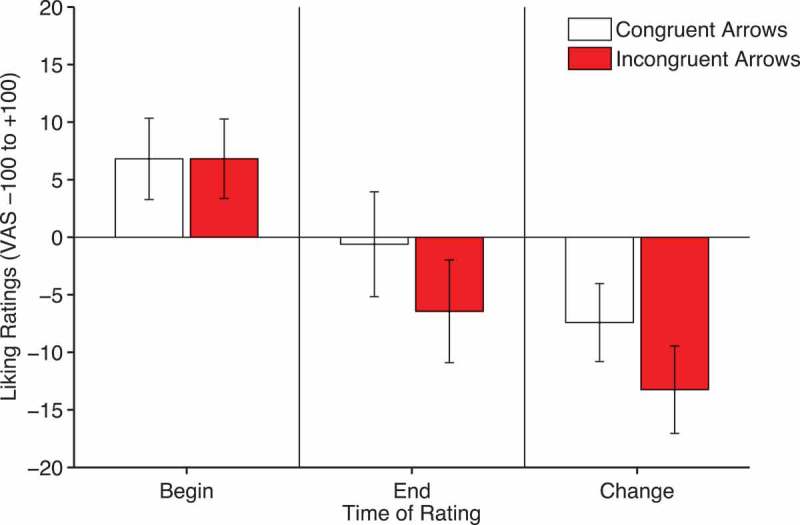
Liking ratings of congruent and incongruent arrows before (left panel) and after (middle panel) cueing and the change in ratings computed by subtracting beginning from end ratings (right panel). Error bars show +/-1 standard error of the mean.

Liking ratings were analyzed with a 2 × 2 within-subjects ANOVA with factors of time and validity. There was a significant effect of time, *F*(1, 30) = 13.39, *p *= .001, *η_p_* ^2^ = .309, owing to generally more negative ratings after (*M = −3*.5, *SEM = *3.4) compared to before cueing (*M = *6.81, *SEM = *3.2). However, there was no significant interaction between time and validity, *F*(1, 30) = 0.942, *p *= .34, *η_p_* ^2^ = .03 (see Figure 9).

#### Arrow-cueing reaction times

**Figure 10.**
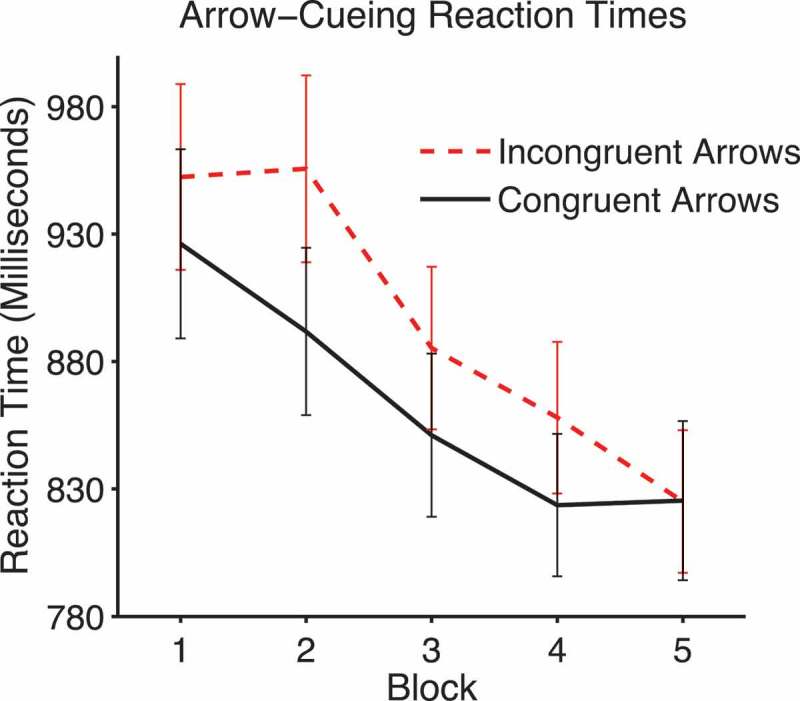
Mean arrow-cueing reaction times on congruent and incongruent trials across blocks. Error bars show +/-1 standard error of the mean.

The arrow-cueing data were analyzed with a 2 × 5 within-subjects ANOVA with factors of validity and block. There was a significant main effect of validity, *F*(1, 30) = 14.02, *p *= .001, *η_p_* ^2^ = .32, due to faster reaction times on congruent (*M = *838.39, *SEM = *51.09) compared to incongruent trials (*M = *874.98, *SEM = *50.86). There was also a significant linear effect of block, *F*(1, 30) = 33.09, *p *< .0001, *η_p_* ^2^ = .524, and a borderline significant interaction of validity with block, *F*(4, 120) = 4.34, *p *= .053, *η_p_* ^2^ = .074 (see Figure 10). This appears to be due to the lack of a cueing effect in Block 5.

Therefore, as noted previously, arrows produce cueing effects that are similar to those evoked by gaze-cues. However, there is no evidence that congruent arrows produce differences in liking ratings compared to incongruent arrows.

### GENERAL DISCUSSION

The current study is the first to use EMG to examine implicit emotional facial reactions to gaze-cues and the relation to individual differences in learning face evaluations of trustworthiness. As in previous studies, reaction times to identify targets were slower when faces gazed away from the target, incongruently, compared to when gazing toward the target, congruently (Driver et al., 1999; Friesen & Kingstone, 1998). Despite the fact that the identity and gaze direction of both incongruent and congruent faces predicted target location, there was still a cueing effect in Block 5. This is consistent with the original study of Driver et al. (1999), which found gaze-cueing effects even when participants knew the face was four times more likely to gaze in the opposite direction to the location of the target. It is also consistent with research showing gaze-cueing effects are immune to manipulations such as backward masking, working memory load, perceptual load, binocular rivalry, and rapid serial visual presentation (Law, Langton, & Logie, 2010; Sato, Okada, & Toichi, 2007; Xu, Zhang, & Geng, 2011).

Also consistent with previous research, incongruent faces that always looked away from targets became judged less trustworthy, while congruent faces that always looked toward targets were subsequently judged more trustworthy (Bayliss et al., 2009; Bayliss & Tipper, 2006) in spite of differences between the dependent measures used across experiments (forced choice vs. ratings)^3^. ^3^In these experiments, we measured trust ratings both before and after cueing as a means of identifying the direction of the effect, whether only congruent increased trust, only incongruent decreased trust, or both. One limitation of this approach is that the initial rating may prime the concept of trust and bias the way in which the stimuli are processed during cueing. It is certainly possible that the initial processing of the faces could produce a stronger representation of identity and hence support the learning of identity-gaze contingencies. However, we note that earlier studies only collected judgments after cueing and still obtained similar effects of gaze on trust (Bayliss et al., 2009; Bayliss & Tipper, 2006). In a second experiment, we demonstrated that this effect of cueing on evaluations appears to be specific to the social qualities of faces and eye-gaze. That is, incongruent arrow-cues did not lead to a difference in ratings of liking compared to congruent arrows despite showing cueing effects that were highly reliable. This corroborates Bayliss, Paul, Cannon, and Tipper (2006), which showed that although gaze and arrows do not differ in their ability to cue attention, objects that are observed to be consistently gazed toward become more liked than those which are gazed away from, an effect which was absent when the cueing stimuli were arrows. These contrasts in learning effects between arrows and faces cannot be explained in terms of differences in visual processing or memory for the stimuli. We compared performance on a preliminary visual search task with arrows and faces and demonstrated that search was equally efficient for both types of stimuli used in our experiments. Of perhaps more importance, we also tested how well arrows and faces were remembered. Clearly linking stimulus identity and behavior (validity of cueing) and retrieving this from memory at a later time is key to the learned trust effects reported here. We observed that recognition memory for the arrows was equivalent to that of faces.

It is important to acknowledge that there are semantic differences between trust and liking which could explain the differences between faces and arrows found in the ratings. Trustworthiness in relation to gaze-cues can be construed in one of two ways. One way it is most commonly understood, and construed in this paper, is in terms of the general willingness to accept risk on another person’s behalf (Rousseau, Sitkin, Burt, & Camerer, 1998). On the other hand, trust can also be construed more specifically in terms of whether one can rely on the gaze-cues to direct the location of the target. The available experimental evidence would seem to suggest that the former construal of trust is more likely learned from gaze-cues. Learning of trust from identity-contingent gaze-cues generalizes from trust judgments to trust behavior in other domains, including in economic exchanges, where participants invest more money with congruent compared to incongruent faces in trust games (Rogers et al., 2014). The fact that arrows showed no learning of liking by cueing congruently or incongruently suggests that arrows either do not evoke emotion and learning or that this learning does not relate to the evaluative cognitive processes of liking. In studies that have asked participants to make preference choices between faces, which had either consistently gazed congruently or incongruently, no significant differences were observed (Bayliss & Tipper, 2006), or differences were weak and inconsistent (Bayliss et al., 2013). Thus, it appears that non-trust-related evaluative adjectives also do not relate to judgments of faces learned from gaze-cues. This suggests a special relationship between faces, gaze, emotion, and trust and that learning is not due to domain-general evaluative conditioning. Indeed, trust is a highly social concept. Although evaluations of faces on a range of dimensions tend to show strong covariation, they can be explained by two underlying dimensions, the strongest of which is most correlated with trust (Oosterhof & Todorov, 2008). However, we do not rule out the possibility that trust could be anthropomorphized to arrow behavior. There are also different types of trust, such as reliability and emotional trust, which can be assessed with different items on a standardized trust questionnaire (Johnson-George & Swap, 1982) in future studies.

Although the changes in trust due to congruent and incongruent gaze-cueing was robust across analysis of all participants, some individuals did not change trust ratings in the expected manner. Hence, we have two groups of participants: Those who increase trust for congruent faces and decrease trust for incongruent faces, and those who show no effects or trends for the opposite assessment of less trust for congruent and more trust for incongruent faces. However, of particular note is the marked similarity of gaze-cueing effects in the two groups of trust and no-trust participants. Hence, it is not simply the impairment of processing produced when a person gazes away from a target object that influences changes in trust ratings. Rather, we proposed something more is required, and that was an emotional reaction, reflected in an embodied state measured via EMG, to the joint attention or deception of gaze-cues.

In an attempt to identify and measure embodied emotional reactions on-line during the gaze-cueing trials we recorded facial EMG from the corrugator brow and zygomaticus cheek muscles. In both the corrugator and zygomaticus muscle there was greater EMG activity during the period when gaze was directed away from target objects on incongruent trials in trial period 4. Previous research has shown that the corrugator is responsive to unpleasant stimuli, whereas the zygomaticus shows a bivalent profile, responding to both pleasant and unpleasant stimuli, albeit somewhat less so to the latter (Armel, Pulido, Wixted, & Chiba, 2009; Lang et al., 1993; Larsen et al., 2003; Sims, Van Reekum, Johnstone, & Chakrabarti, 2012). However, although the zygomaticus response was sensitive to gaze-cueing it did not differentiate between the trust and no-trust participants. As identifying what factors might underlie these individual differences is the central focus of this work, we will not discuss the zygomaticus data further.

The corrugator muscle response produces an intriguing pattern of data revealing individual differences in learning of trust. On the one hand it confirms our hypothesis that it is embodied emotional responses detected via EMG that determine the learning of trust via identity-contingent gaze-cues. That is, those who produce the expected change in trust ratings, where incongruent faces are trusted less than congruent faces, produce a different EMG pattern than those who do not show this trust effect. Notably, during trial period 4, there is a significantly greater EMG response in the corrugator to faces whose gaze is incongruent with respect to the target, but only in those people who later show less trust of these faces compared to congruent faces. Figure 6 (Panels E & F) reveals that this pattern was consistent throughout the experiment. The contrast in EMG response between trust and no-trust individuals was the pattern predicted by the hypothesis that embodied emotional responses were critical for learning about trust.

During trial periods 2 and 3 the contrast between congruent and incongruent faces is not present in the stimulus array. During trial period 2 the face looks straight ahead, and during trial period 3, although a gaze shift to right or left has taken place, the target is not yet present, and hence it is not known whether this will be a congruent or incongruent cueing trial. Therefore, any contrasts between congruent and incongruent faces must be due to retrieval of prior episodes from memory. Figure 6 (Panels B & D) shows this learning process. During the first block of trials where learning has not taken place, there is no contrast between congruent and incongruent faces. However, as the experiment progresses and there is continued exposure to the faces and whether they consistently look toward or away from targets, the discrimination between congruent and incongruent faces increases, just as a learning mechanism would predict.

**Figure 11.**
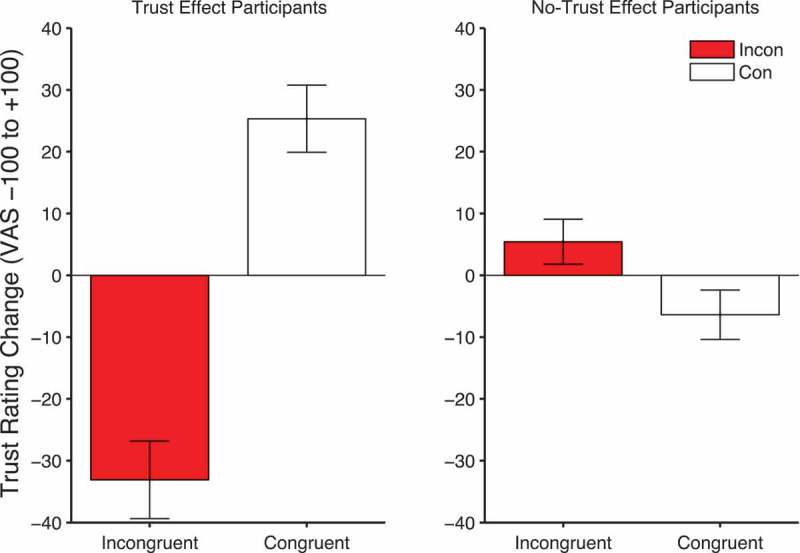
Mean change in trust ratings (end-beginning) for congruent and incongruent conditions in trust effect (left panels) and no-trust effect participants (right panels). Error bars show +/-1 standard error of the mean.

However, two aspects of the trial period 2 and trial period 3 data were not predicted. Firstly, the retrieval of gaze-identity contingencies is only significant in those individuals who did not produce our standard trust effect. Secondly, the effect is in the opposite direction: There is greater corrugator response to congruent than incongruent faces. This suggests that these participants have more negative embodied reactions when viewing faces that will shortly look toward subsequent targets. Figure 11 shows the change in trust ratings for both those participants who show the standard trust effect (left panel) and those who do not (right panel). Intriguingly, the no-trust participants produce a significant time × validity interaction (*F*(1, 22) = 5.98, *p *= .023, *η_p_* ^2^ = .214) in the opposite direction, where trust of incongruent faces increases and trust of congruent faces slightly declines. Hence, the corrugator response during trial period 2 and trial period 3 matches these later trust ratings.

Unfortunately, we only collected individual difference measures of anxiety and no effects were identified there. Therefore, we cannot explain what personality types might find individuals who consistently look toward targets and facilitate responses less trustworthy than individuals who consistently look in the wrong direction. It is possible that measures of empathy, autism, schizotypy, or depression might map on to the embodied EMG and trust ratings measures. Certainly deficits of theory of mind and processing of social stimuli such as gaze-cues have been proposed as factors in autism (Campbell et al., 2006), schizophrenia (Roux, Forgeotd’Arc, Passerieux, & Ramus, 2014), and perhaps depression. Indeed, our unpublished data has compared individuals with depression to matched controls in similar gaze-cueing tasks. Although both populations produced similar gaze-cueing effects, those with depression tended to respond similarly to the no-trust population in the current study, with a small trust preference for the incongruent faces (Rogers, Bayliss, Wakeley, Cowen, & Tipper, unpublished manuscript). Recent work has also provided evidence for the role of embodied emotions in depression. Injections of botulinum toxin into the corrugator muscle suppresses its activity and inhibits embodied emotional feedback reducing symptoms of depression (Wollmer et al., 2012). Clearly, recording of embodied emotions via EMG in these populations would be worthwhile.

While the current study identifies a role for embodied emotion in mediating the learning of trust judgments from gaze-cues, it is a matter of debate as to what aspect of cueing is responsible for triggering the emotion. Possible explanations come in the form of fluency, the reinforcement value of shared attention, and the association between gaze-cues and deception. In terms of fluency, the deleterious effects of incongruent gaze-cues on reaction times may introduce dysfluency into attentional orienting, stimulus categorization, and response selection when compared with congruent cues. It is well established that fluency of perceptual processing influences emotional responses during judgments of preference (Reber, Winkielman, & Schwarz, 1998; Zajonc, 1968). However, the current data do not support the fluency account, as the gaze-cueing facilitation effects are similar in those who do and do not produce later changes in trust ratings. Similarly, as noted above, both gaze-cues and arrows produce similar effects on fluency in terms of facilitation, but only gaze-cues change liking of objects (Bayliss et al., 2006) and affective evaluations of cue identity. Hence, there has to be something more than simple speed of response fluency that only faces possess.

A more likely possibility is that there may be reduced reinforcement when gaze is directed away from an object and the participant is required to break away from shared attention to attend to the opposite side of the screen. Indeed, Schilbach et al. (2010) found that there was increased activity in the ventral striatum to shared compared to non-shared attention, reflecting reduced reinforcement signals.

The findings of the current study may also be related to the use of gaze-cues for deception. There have been several descriptions of the way in which primates and humans can use their own social-attention cues to misdirect the attention of others and further their own interests (Allison, Puce, & McCarthy, 2000; Emery, 2000; Klein, Shepherd, & Platt, 2009; Whiten & Byrne, 1988). For example, skilled basketball players can reliably misdirect a defending player’s attention from the direction of the pass by orienting their head in the opposite direction (Kunde, Skirde, & Weigelt, 2011). Thus, the incongruent gaze-cues may be perceived as if the actor is intentionally trying to misguide the participant’s attention. This intentionality is clearly signaled by the fact that the actor chooses to look away from a highly salient/interesting object in the absence of any competing stimuli.

Clearly, our current data show that the reinforcement value of joint attention or detection of deception are not necessarily the same in all participants. It will be important to consider converging techniques to examine these processes. As we have noted, gaze-cueing effects do not differentiate between trust/no-trust participant groups whereas EMG does. In a separate study, EEG was used to measure the late positive potential (LPP), which is related to emotion processing (Manssuer et al., 2015). This measure did not detect differences between participants who produced trust and no-trust effects. However, we note that the LPP and corrugator EMG reflect qualitatively different aspects of the emotional response. Cuthbert, Schupp, Bradley, Birbaumer, and Lang (2000) measured the LPP, corrugator EMG, and valence and arousal ratings in response to positive, negative, and neutral pictures and submitted them to factor analysis. They found that the LPP and arousal ratings both loaded onto a factor that was separate to a second factor loaded onto by corrugator EMG and valence ratings. Thus, the LPP is more related to emotional arousal and corrugator EMG is more reflective of the emotional valence of the evoking stimulus.

It is also essential to examine the neural systems mediating these differences. For example, the STS and striatum discriminate gaze toward versus gaze away from objects, the former being concerned with the shift of attention via links to the parietal lobe (e.g., Pelphrey, Morris, & McCarthy, 2005), while the latter encodes the reinforcement value of stimuli. We predict that our trust and no-trust participants may show similar responsiveness in the STS to congruent and incongruent trials, as the shift of attention evoked by gaze-cues are similar. In contrast, it may be the higher-level interpretation and emotional response to observed gaze behavior that may differ in structures such as the striatum.

In conclusion, here we provide initial evidence from facial EMG for the role of embodied emotion and individual differences in learning face evaluations of trustworthiness from gaze-cueing. We identified two populations. One group showed the standard trust effect whereas the other did not. The shift of attention, as measured by gaze-cueing effects did not discriminate between these groups. In sharp contrast, measures of embodied emotion via facial EMG did discriminate these groups. However, the data did not support our simple starting hypothesis that only those whose EMG responses discriminated between congruent and incongruent faces would learn about trust. Rather we found two distinct patterns of EMG response in the trust and no-trust populations that discriminated congruent and incongruent faces. Future research should specifically focus on understanding why the no-trust group appeared to find congruent faces that looked toward targets less trustworthy in their ratings and had increased negative responses as reflected by corrugator activity.
